# Gene Expression Analysis of Potato (*Solanum tuberosum* L.) Lipoxygenase Cascade and Oxylipin Signature under Abiotic Stress

**DOI:** 10.3390/plants11050683

**Published:** 2022-03-02

**Authors:** Svetlana Gorina, Anna Ogorodnikova, Lucia Mukhtarova, Yana Toporkova

**Affiliations:** Kazan Institute of Biochemistry and Biophysics, FRC Kazan Scientific Center of RAS, P.O. Box 30, 420111 Kazan, Russia; anyuta_ogorodnik@mail.ru (A.O.); lucia74@yandex.ru (L.M.)

**Keywords:** potato (*Solanum tuberosum* L.), lipoxygenase pathway, CYP74 family, abiotic stress

## Abstract

The metabolism of polyunsaturated fatty acids through the lipoxygenase-catalyzed step and subsequent reactions is referred to as the lipoxygenase (LOX) pathway. The components of this system, such as jasmonates, are involved in growth, development and defense reactions of plants. In this report, we focus on dynamics of expression of different LOX pathway genes and activities of target enzymes with three abiotic stress factors: darkness, salinity and herbicide toxicity. To obtain a more complete picture, the expression profiles of marker genes for salicylic acid, abscisic acid, ethylene, auxin and gibberellin-dependent signaling systems under the same stresses were also analyzed. The gene expression in *Solanum tuberosum* plants was analyzed using qRT-PCR, and we found that the LOX-cascade-related genes responded to darkness, salinity and herbicide toxicity in different ways. We detected activation of a number of 9-LOX pathway genes; however, in contrast to studies associated with biotic stress (infection), the 9-divinyl ether synthase branch of the LOX cascade was inhibited under all three stresses. GC-MS analysis of the oxylipin profiles also showed the main activity of the 9-LOX-cascade-related enzymes after treatment with herbicide and darkness.

## 1. Introduction

Due to the lack of the capability to move as a means of reacting to changes in their environment, plants have to adapt to environmental stresses in other ways. Oxylipin metabolism is one of the plant defense mechanisms. Oxylipins are a constantly growing group of low-molecular-weight compounds that comprise oxygenated fatty acids and their derivatives [1]. The main source of oxylipins is the lipoxygenase (LOX) cascade. The most studied oxylipins are jasmonates, products of the allene oxide synthase (AOS) branch of the LOX cascade. Among jasmonates, jasmonoyl-isoleucine (JA-Ile) mediating the plant cell response to biotic and abiotic stresses is the most studied [2]. Besides JA-Ile, the direct effect of a number cyclopentenones (10-oxo-11-phytodienoic acid (10-OPDA), 10-oxo-11-phytoenoic acid (10-OPEA)) on the growth of fungi and herbivorous insects has been described [3,4]. In addition, there is a group of active airborne metabolites such as methyl JA and *cis*-jasmone, which act as messengers between plants to transmit signals and activate defense genes [5]. Other well-studied oxylipins are green leaf volatiles (GLVs), composed of C6 aldehydes, alcohols and their esters, formed through the hydroperoxide lyase (HPL) branch of the LOX pathway. These compounds have been shown to play key roles in plant–plant and plant–herbivore interactions [6,7]. Additionally, they function as signals activating systemic defense [8,9], and are involved directly in plant defense as antimicrobials. For example, (2*E*)-hexenal [10] and (3*E*)-hexenal [11] possess antibacterial properties, while (2*E*)-nonenal and (2*E*)-hexenal exhibit antifungal properties inhibiting hyphal growth [12]. Besides GLVs, the HPL pathway results in formation of traumatic acid, which is involved in wound healing [13].

In addition to AOS and HPL branches, there are divinyl ether synthase (DES) [14,15,16] and epoxyalcohol synthase (EAS) branches [17] of the LOX pathway. These branches are less studied. The literature describes the participation of divinyl ethers in protection against a number of pathogens: bacteria [18], oomycetes [19] and fungi [20]. The cloning and characteristics of several AOSs, HPLs, DESs and EASs from different plant species revealed that they are closely related members of the cytochrome P450 family, and form the CYP74 family, which is specialized in the metabolism of hydroperoxides [21].

The potato (*Solanum tuberosum* L.) is the most important non-cereal nutritional crop in the world [22], and belongs to the Solanaceae family, which also includes a number of other agricultural crops (tomato, pepper, eggplant), medicinal plants, spices and ornamentals [23]. Potatoes originated in the Andean mountain region of South America, and now there are two main cultivar groups of *Solanum tuberosum: andigena* or Andean; and *tuberosum* or Chilean [24]. Cultivated potato species have a base chromosome number of n = 12, and can be of different ploidy levels: di-, tri-, tetra- or pentaploid [23].

In this work, we focused on the study of gene expression of the lipoxygenase pathway of potato and other related hormone (auxin, ethylene, abscisic acid, salicylic acid and gibberellin) signaling systems during abiotic stresses. Along with this, the activity of enzymes of the LOX pathway was assessed in the analyzed samples. Our results demonstrate that different types of abiotic stress are characterized by their own pattern of expression of genes of the LOX cascade, as well as by different activities of target enzymes (LOX, CYP74, etc.). Significant changes in oxylipin profiles were associated with the 9-LOX pathway of linoleic acid metabolism. However, in contrast to studies associated with biotic stress (infection), the 9-DES branch of the LOX cascade was inhibited during darkness, salinity and herbicide toxicity.

## 2. Results and Discussion

### 2.1. Bioinformatic Analysis of S. tuberosum CYP74 Sequences

**Figure 1:**
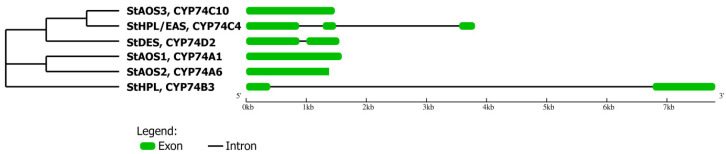
Phylogenetic tree and gene structure of the CYP74 members of *S. tuberosum*. The green areas represent exons; lines show the introns.

There are six CYP74 genes in the potato genome, including one DES gene (StDES), one HPL gene (StHPL) and one dual HPL/EAS (StHPL/EAS), as well as three AOS genes (StAOS1, StAOS2 and StAOS3). Based on the NCBI and Phytozome data, the exon–intron structure and localization of potato CYP74 genes was established. AOS genes are localized on the following chromosomes: StAOS1: on the 4 chr; StAOS2: on the 22 chr; and StAOS3: on the 10 chr. The StDES gene is localized on the 1 chr, the StHPL gene is localized on the 7 chr and the StHPL/EAS gene is localized on the 1 chr. AOS genes (StAOS1, StAOS2 and StAOS3) do not have introns; both StHPL and StDES genes have one intron and StHPL/EAS gene has two introns (Figure 1).

The CYP74 enzymes of potato belong to four subfamilies (A, B, C and D), and are classified as follows: CYP74A1 (StAOS1); CYP74A6 (StAOS2); CYP74C10 (StAOS3); CYP74C4 (StHPL/EAS); CYP74B3 (StHPL); and CYP74D2 (StDES). We built a phylogenetic tree to assess the similarity of the CYP74 enzymes (Figure 1). As expected, the CYP74A and CYP74C enzymes group with each other. CYP74D2 is located closer to the CYP74C members, while CYP74B3 is located separately from other enzymes.

### 2.2. Cis-Element Analysis of S. tuberosum CYP74 Genes

**Figure 2:**
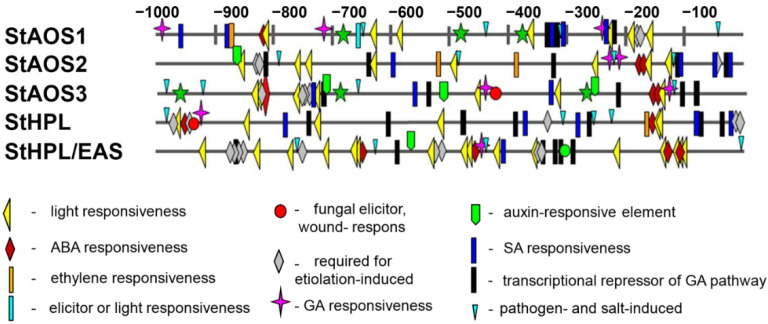
Distribution of *cis*-elements identified in *S. tuberosum* CYP74 genes. Different colors and shapes represented different *cis*-acting elements, which are most enriched in the promoter regions up to −1000 from CYP74 genes.

To assess the possible transcriptional regulation of potato CYP74 genes, 1000 bp sequences upstream of the translation start site were scanned by PlantCARE and PLACE. Various regulatory elements were found in the promoter regions of the CYP74 genes, among which the widely represented were the following elements: involved in light responsiveness (such as Box 4, G-Box, GT1-motif, TCT-motif, MRE, I box, SORLIP1AT), involved in the abscisic acid responsiveness (ABRE), required for etiolation-induced expression (ACGTATERD1, ABRELATERD1) and playing a role in responses to pathogens and salinization (GT1GMSCAM4) (Figure 2). One to six (depending on the gene) salicylate-responsive elements (TCA-element, WBOXATNPR1), auxin-responsive elements (TGA-element, NTBBF1ARROLB, gibberellin-responsive elements (MYB, P-box, GARE1OSREP1) and transcriptional repressor of the gibberellin pathway (WRKY71OS) (Figure 2) were also detected. *Cis*-elements, their sequences and the corresponding database are shown in Table 1. Thus, the analysis of regulatory elements carried out showed that the promoter regions of genes containing *cis*-acting elements responded to abiotic stresses, light and phytohormones, etc.

### 2.3. Expression Analysis of Marker Genes of Lipoxygenase Cascade and Other Signaling Systems of S. tuberosum after Growing in the Dark

To investigate the dynamics of expression pattern of genes of lipoxygenase cascade (Supplementary Table S1) in potato plants in conditions of darkness, salinity and herbicide toxicity, we monitored mRNA levels in shoots and roots in different time intervals after treatments by qRT-PCR. Etiolation involves prolonged growth in the absence of light, resulting in the formation of etioplasts, structures which do not contain chlorophyll or stacked thylakoid membranes, and the lipid composition of which consists mainly of monogalactosyldiacylglycerol (MGDG) and digalactosyldiacylglycerol (DGDG) [25,26]. Additionally, as was shown earlier in Arabidopsis [27], during etiolation, the content of unsaturated C18 fatty acids (oleic (18:1), linoleic (18:2) and α-linolenic (18:3) acids) in cutin polymer of etiolated hypocotyls increases. In higher plants, the main substrates of the lipoxygenase cascade are linoleic and α-linolenic acids.

**Figure 3:**
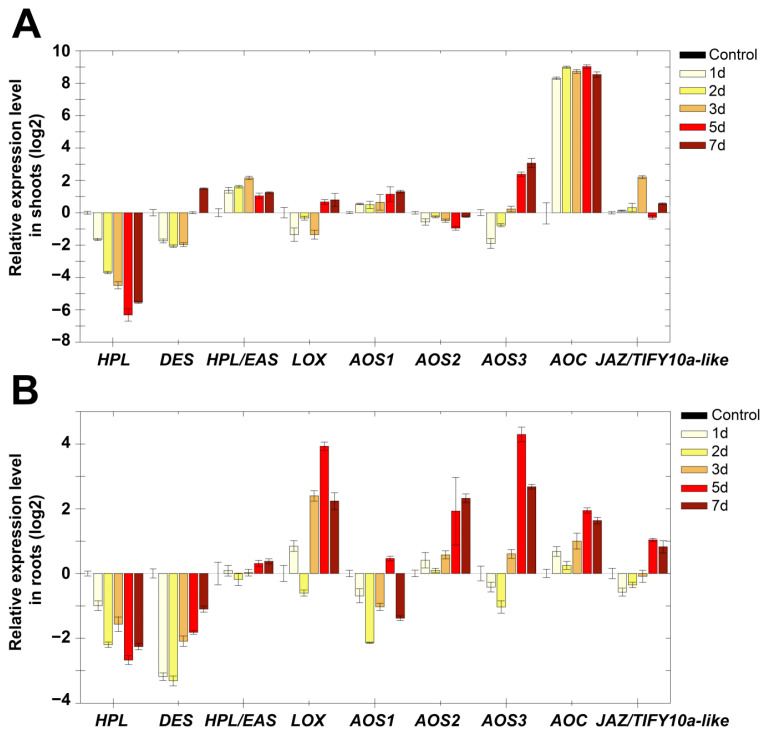
Dynamics of lipoxygenase cascade gene expression (log2) during darkness in shoots (**A**) and roots (**B**) in 1, 2, 3, 5 and 7 days. Abbreviations: HPL: hydroperoxide lyase, DES: divinyl ether synthase, HPL/EAS: hydroperoxide lyase/epoxyalcohol synthase, LOX: lipoxygenase, AOS: allene oxide synthase, AOC: allene oxide cyclase and JAZ/TIFY10a-like: JAZ/TIFY transcription regulator gene.

Darkness of potato plants led to a gradual pronounced decrease in *StHPL* expression by two orders of magnitude in shoots and by one order of magnitude in roots compared to the control (Figure 3). Expression of *StDES* had a similar pattern. The expression of *StHPL/EAS* slightly (2–4 times) increased in shoots, and remained at the control level in roots. *StAOS3* expression gradually increased, and in 7 days it differed by 10 times from the control, both in shoots and roots. Expression pattern of *StAOS1* and *StAOS2* were opposite: expression of *StAOS1* slightly increased in shoots and decreased in roots, while expression of *StAOS2* decreased in shoots and increased in roots. Apparently, a switch between two AOS branches occurred. Along with this, *StLOX* expression increased only in roots, while in shoots it remained comparable with control values. It is interesting that the most pronounced increase in shoots was observed for allene oxide cyclase (StAOC) by almost three orders of magnitude in all analyzed samples. In roots, *StAOC* expression also increased. It was previously shown that an increase in the AOC gene expression level can be induced by wounding [28]; however, the accumulation of AOC mRNA may not lead to the accumulation of jasmonates [29,30], and as a consequence, the appearance of a JA response. Apparently, along with the regulation of JA signaling at the level of signal transduction, there is a regulation mechanism through a limiting substrate. This mechanism activates expression of upstream genes of the JA biosynthetic pathway, such as AOC, to provide a sufficient amount of substrate for a quick response if needed. The greatest increase in AOC expression in shoots is consistent with the previously established existence of JA transport (or its precursors) from shoots to roots [31].

The expression of the allene oxide cyclase (StAOC) encoding gene is greatly increased in the absence of AOS gene expression activation, and requires separate consideration. Apparently, the cyclase reaction in this case is limiting in the biosynthesis of jasmonic acid, while AOS forms a large amount of allene oxide regardless of the presence or absence of stress. However, since allene oxide is a short-lived product that cannot be deposited, in the absence of stress (in the absence of a sufficient amount of cyclase), all excess allene oxide is hydrolyzed to α-ketol.

Apparently, one can observe switching from the HPL and DES branches of lipoxygenase cascade to AOS ones, wherein AOSs are participants of different branches producing different compounds. StAOS1 and StAOS2 catalyze the formation of allene oxide 12,13-EOT ((9*Z*,11*E*,13*S*,15*Z*)-12,13-epoxy-(9,11,15)-octadecatrienoic acid), a precursor of jasmonic acid, while StAOS3 is a participant in 9-lipoxygenase branch products, which include ketols and cyclopentenones. Patterns of expression of AOS genes indicate that in the shoots, the jasmonate pathway (13-LOX branch) is activated, while in roots, both 9-LOX and 13-LOX pathways are activated.

**Figure 4:**
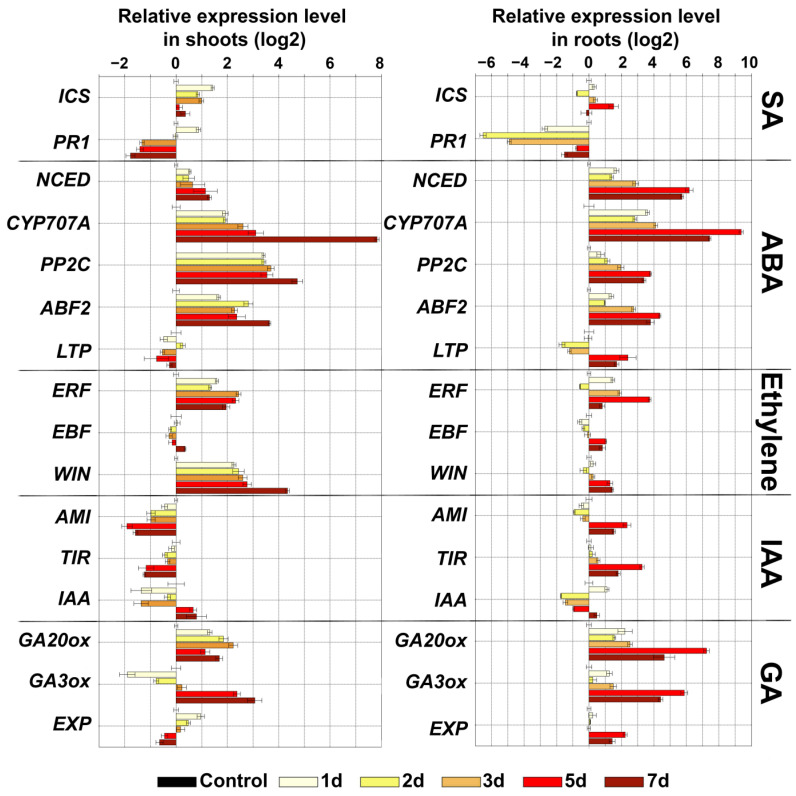
Changes of expression of marker genes of different signaling pathways during darkness in shoots and roots in 1, 2, 3, 5 and 7 days. Abbreviations: ICS: isochorismate synthase, PR1: pathogenesis-related protein 1, ERF: ethylene-inducible transcription factor, EBF: EIN3 (ETHYLENE-INSENSITIVE3) BINDING F-BOX1, WIN: wound-induced (WIN) protein, NCED: 9-*cis*-epoxycarotenoid dioxygenase, CYP707A: ABA-8′-hydroxylases, ABF2: ABRE-binding bZIP proteins, PP2C: serine/threonine phosphatase 2C, LTP: lipid transfer protein, GA20ox: gibberellin (GA) 20-oxidase, GA3ox: GA3 oxidase, EXP: expansins, AMI: indole-3-acetamide hydrolase, TIR: transport inhibitor response and IAA: indole-3-acetic acid inducible 28 (IAA28).

Since in plant cells, crosstalk between lipoxygenase cascade and other signaling pathways (auxin (indole-3-acetic acid, IAA), gibberellin (GA), ethylene (ET), abscisic acid (ABA) and salicylic acid (SA)) occurs [32,33,34,35,36], the expression of a number of marker genes (Supplementary Table S2) of other signaling pathways was analyzed under the same conditions. Analysis of the expression of marker genes for 7 days during darkness showed that the pattern of gene expression was comparable in shoots and roots (Figure 4). It was shown that darkness led to a slight accumulation of transcripts of the isochorismate synthase (ICS) gene, which is involved in the biosynthesis of salicylic acid. The level of transcripts of this gene was increased compared to the control in shoots up to 2.6 times, and in roots up to 2.8 times. The incubation of plants in the dark induced a gradual accumulation of most ABA marker genes both in shoots and roots. The following changes were observed in roots: 9-*cis*-epoxycarotenoid dioxygenase (NCED, ABA biosynthesis) (from 0 to 72 times), ABA-8′-hydroxylases (CYP707A, ABA degradation) (from 0 to 666 times), serine/threonine phosphatase 2C (PP2C, negative regulator of ABA signal transmission) (from 0 to 13 times), as well as another important participant in ABA signaling, a transcription factor ABF2 belonging to the ABRE-binding bZIP proteins designated as ABF family (from 0 to 20 times). The shoots had the same pattern, but with slightly less pronounced dynamics. In contrast to those described above, only the lipid transfer ABA-dependent protein (LTP) coding gene was characterized by a lower level of expression up to 0.5-fold in shoots, and a gradual increase from 0.3- to 5-fold compared to the control.

One of the defense mechanisms is associated with ethylene, since expression of genes of ethylene-inducible transcription factor (ERF) and a wound-induced (Win) protein, which also has an ethylene-dependent character of transcription [37], increased. Expression of the gene of the negative regulator of ethylene-dependent signaling *EBF* (EIN3 (ETHYLENE-INSENSITIVE3) BINDING F-BOX1) remained comparable to the control. Growth of plants in the dark, as expected, led to an increase in the expression of genes encoding enzymes of gibberellin biosynthesis (GA3 oxidase (GA3ox), GA20-oxidases (GA20ox)). The darkness did not significantly affect the expression of the EXP gene. The level of transcripts of this gene gradually decreased in shoots from 1.9 to 0.6 times, and increased from 1 to 4 times in roots compared to the control. Additionally, the expression level of indole-3-acetamide hydrolase (AMI, auxin biosynthesis) and transport inhibitor response (TIR1) genes in shoots gradually decreased from 0.7 to 0.25 and from 0.9 to 0.4, respectively. The expression level of the repressor of IAA signaling (IAA28) gene gradually increased from 0.3 to 1.7 times. In the roots, the expression pattern was reversed. The level of transcripts increased from 0.71 to 5 and from 1 to 9 for *AMI* and *TIR1*, respectively. The expression level of *IAA28* decreased to 0.3.

Thus, growing potato plants in the dark is associated with the activation of the biosynthesis of jasmonates and gibberellins, as well as the biosynthesis and active utilization of ABA. The presence of ABA in cells is indirectly evidenced by the accumulation of transcripts of PP2C and ABF2 genes, which are both ABA-inducible. These results are consistent with the data on the effect of ABA promotion on the growth of etiolated tomato seedlings [38]. The function of ABA is dose-dependent, and ranges from stimulatory to inhibitory effects [39]. Thus, the accumulation of PP2C gene transcripts indicates a fine-tuned regulation of the ABA content.

The absence of light also led to the accumulation of gibberellins [40], which in this study is evidenced by the accumulation of transcripts of GA3ox and GA20ox genes of gibberellin biosynthesis. Additionally, the relationship between not only ABA and GA but also GA and JA was established. Moreover, these relationships exhibited both antagonistic and synergistic interactions [41,42,43]. Thus, a significant accumulation of GA20ox and GA3ox transcripts (by one to two orders of magnitude relative to the control), along with the predominance of gene expression of the 9-LOX pathway over the 13-LOX pathway (Figure 3), testifies to the GA response and inhibition of JA. Changing patterns of gene expression indicate complex cross-signaling required for the adaptation and growth of plants in darkness.

### 2.4. Expression Analysis of Marker Genes of Lipoxygenase Cascade and Other Signaling Systems of S. tuberosum during Oxidative Stress

To investigate the role of lipoxygenase cascade enzymes during oxidative stress, reactive oxygen species (ROS) generation was induced by the addition of 10^−5^ and 10^−6^ M paraquat. Paraquat is one of the most widely used non-selective herbicides. It is a potent inducer of oxidative stress, leading to an increase in ROS production, as well as suppressing the regeneration of reducing equivalents and compounds required for the antioxidant system [44].

**Figure 5:**
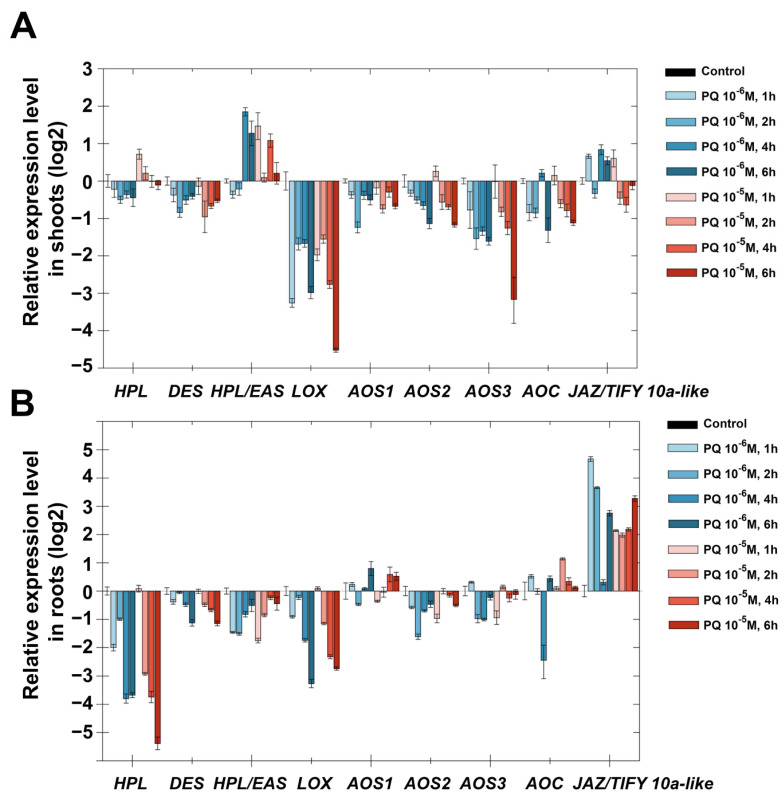
Dynamics of lipoxygenase cascade gene expression after treatment with paraquat (PQ) (10^−5^ (red) and 10^−6^ (blue) M) in shoots (**A**) and roots (**B**) in 1, 2, 4 and 6 h. Abbreviations: HPL: hydroperoxide lyase, DES: divinyl ether synthase, HPL/EAS: hydroperoxide lyase/epoxyalcohol synthase, LOX: lipoxygenase, AOS: allene oxide synthase, AOC: allene oxide cyclase and JAZ/TIFY10a-like: JAZ/TIFY transcription regulator gene.

The results showed that treatments with paraquat in both concentrations (10^−5^ and 10^−6^ M) in shoots and roots did not affect or lead to a decrease in the expression level of most genes of the lipoxygenase cascade Figure 5. The exception was the JAZ/TIFY transcription regulator gene (*JAZ/TIFY10a-like*), the expression of which increased in roots by an order of magnitude at both concentrations of paraquat compared to the control. In literature, this class of regulators is described as negative regulators of jasmonate signaling that repress transcription factors such as MYC2 [45,46]. However, their expression increases upon the infection of plants [47], which is associated with the strong activation of the jasmonate response and the subsequent activation of the JAZ/TIFY genes. It was shown that abiotic stress also affects the increase in JAZ/TIFY expression [48], including the studied TIFY10a [49]. Thus, the transgenic *Medicago sativa* plants overexpressing *GsTIFY10a* (*Glycine soja*) gene grows better under alkaline stress [49], since overexpression of *GsTIFY10a* promoted an increase in the expression of genes necessary for maintaining homeostasis, cytoplasmic pH regulation and osmotic regulation (*NADP-ME, H^+^-ppase, P5CS*) [49,50]. In the present work, an increase in the expression of *JAZ/TIFY10a-like* may indicate both a general response to abiotic stress and suppression of the jasmonate response, since other genes of jasmonate biosynthesis are also down-regulated.

**Figure 6:**
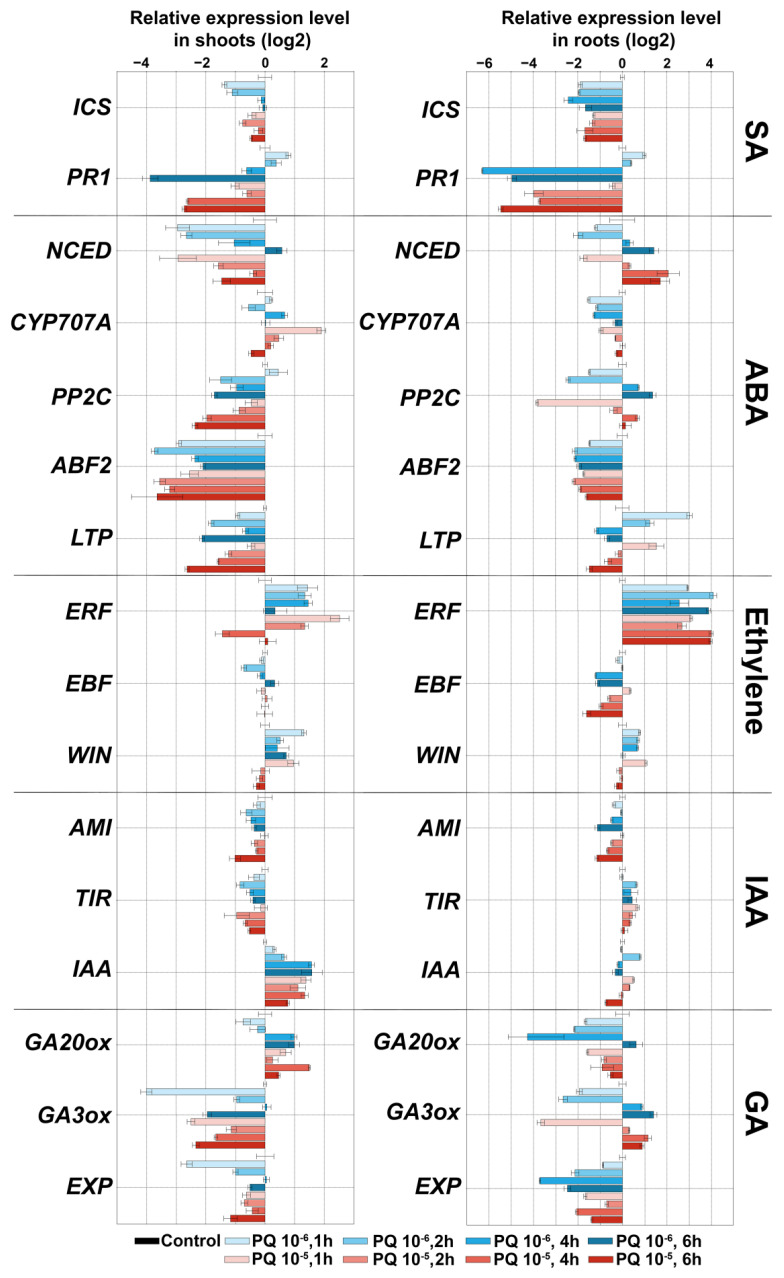
Changes in marker gene expression of other signaling pathways after treatment with paraquat (PQ) (10^−5^ M (red) and 10^−6^ M (blue)) in shoots and roots in 1, 2, 4 and 6 h. Abbreviations: ICS: isochorismate synthase, PR1: pathogenesis-related protein 1, ERF: ethylene-inducible transcription factor, EBF: EIN3 (ETHYLENE-INSENSITIVE3) BINDING F-BOX1, WIN: wound-induced (WIN) protein, NCED: 9-*cis*-epoxycarotenoid dioxygenase, CYP707A: ABA-8′-hydroxylases, ABF2: ABRE-binding bZIP proteins, PP2C: serine/threonine phosphatase 2C, LTP: lipid transfer protein, GA20ox: gibberellin (GA) 20-oxidase, GA3ox: GA3 oxidase, EXP: expansins, AMI: indole-3-acetamide hydrolase, TIR: transport inhibitor response and IAA: indole-3-acetic acid inducible 28 (IAA28).

The expression pattern of marker genes after treatment with paraquat (Figure 6) was different from that during darkness. In shoots and roots, the number of ICS gene transcripts was reduced in all samples. In roots, the NCED gene transcripts gradually accumulated in 6 h after treatment (up to 4 times), followed by the accumulation of the PP2C gene transcripts (up to 2 times), while the LTP gene expression decreased (from 8 times in 1 h to values below the control by 6 h). Expression of two other marker genes of ABA signaling (CYP707A and ABF2) decreased in roots in all experimental samples. In shoots, the expression of almost all ABA marker genes was lower than in the control. Thus, the expression of the NCED gene in the first hour after treatment was lower than in the control by an order of magnitude, and only in 6 h after treatment reached the level of control value. The expression of PP2C, ABF2 and LTP genes was stably lower than in the control, and CYP707A gene expression was comparable to the control.

The expression pattern of ethylene marker genes after the treatment of paraquat was similar to that during darkness: *ERF* and *WIN* expression increased and *EBF* expression was comparable to the control in shoots, and decreased in the roots up to 0.3 times. Expression of *AMI* and *TIR1* genes decreased in shoots up to 0.4 and 0.5 times, respectively, while *IAA28* gene expression slightly increased up to 2.5 times. In the roots, the number of transcripts of *AMI* was also reduced by 0.4 times, while *TIR1* increased to 1.5 times and *IAA28* decreased from 1.7 to 0.5 times. The expression of genes of GA biosynthesis, in contrast to the darkness, did not have a definite pattern, and fluctuated depending on the experimental point. In the shoots, the expression of the *GA20ox* gene increased from 0.6 to 2 times, while the expression of the *GA3ox* gene decreased up to 0.06 times (at PQ 10^−6^ M) and up to 0.15 times (at PQ 10^−5^ M). In the roots, on the contrary, the expression of GA20ox gene decreased (up to 0.2 times), while the GA3ox gene expression had a slight upward trend (up to 2.5 times) in 14 days. The EXP gene expression was reduced in all samples compared to the control plants.

ABA is one of the main phytohormones involved in defense responses to drought, salt, osmotic or cold stress [51,52]. Paraquat treatment also led to changes in the expression of ABA marker genes (NCED, CYP707A, PP2C, ABF2 and LTP) (Figure 6). In the roots, there was a slight activation of the ABA biosynthesis gene (NCED) from 0.4 to 4.5 times. The number of NCED transcripts in the shoots was reduced by an order of magnitude (up to 0.1 times). The expression of genes with an ABA-dependent expression pattern in shoots was also reduced, from PP2C to 0.2, ABF2 to 0.07 and LTP to 0.15 times, respectively. Along with this, activation of transcription of the CYP707A gene was observed (up to 3 times, degradation of ABA). In the roots, along with a decrease in the expression of the ABF2 gene (up to 0.2 times), the number of transcripts of the PP2C gene gradually increased from 0.3 to 2.5, while the number of the LTP gene, on the contrary, decreased from 8 to 0.3 times. The results obtained, along with the activation of ABA biosynthesis genes, indicated a possible inhibition of ABA signal transduction. The picture of ABA signaling in shoots and roots most likely differed, since the patterns of gene expression did not match.

A clearer picture was observed for ethylene. An increase in the expression of *ERF* and *WIN* (in shoots and roots) indicated the activation of ethylene-dependent genes, which is consistent with the data that ET signaling and biosynthesis are induced under various agents causing oxidative stress [53]. In this case, the expression of ERF genes is important for signaling and expression of genes of the antioxidant system [54,55]. The expression of auxin-related and GA-related genes was comparable to the control. As a whole, ET signaling was activated and SA and JA signaling inhibited. GA and auxin signaling did not differ from control.

### 2.5. Expression Analysis of Marker Genes of Lipoxygenase Cascade and other Signaling Systems of S. tuberosum after Treatment with NaCl

Salt stress not only has a negative effect on biochemical processes in plants, but also reduces their agricultural value. Moreover, the salinity toxicity problems are gradually increasing around the world [56]. In this regard, the present work on study the effect of NaCl-induced salt stress on expression of marker genes of lipoxygenase cascade and other signaling systems is of current interest. The results showed that the expression profile of target genes in shoots and roots had differences.

**Figure 7:**
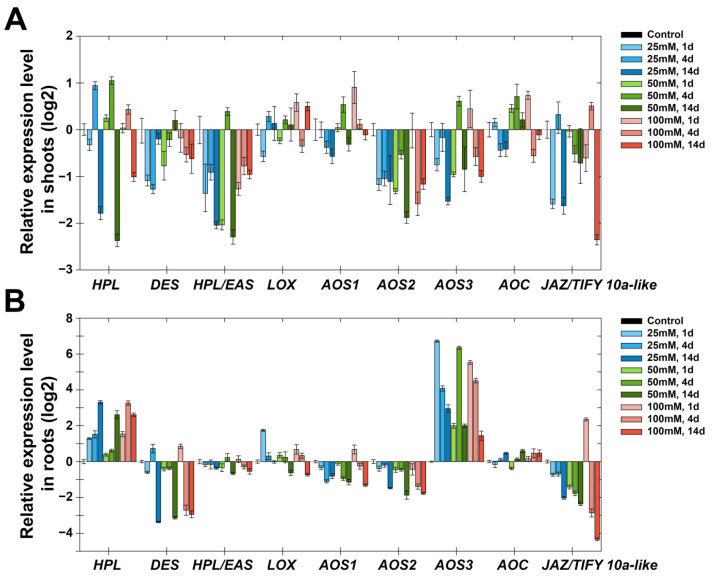
Expression of lipoxygenase cascade genes in response to treatment with NaCl (25, 50, 100 mM) in shoots (**A**) and roots (**B**). The blue color indicates the concentration of 25, while green denotes 50 and red 100 mM. The color gradation indicates samples at 1, 4 and 14 days. Abbreviations: HPL: hydroperoxide lyase, DES: divinyl ether synthase, HPL/EAS: hydroperoxide lyase/epoxyalcohol synthase, LOX: lipoxygenase, AOS: allene oxide synthase, AOC: allene oxide cyclase and JAZ/TIFY10a-like: JAZ/TIFY transcription regulator gene.

Treatment with NaCl (25, 50 and 100 mM) led to a decrease in the expression of *StDES*, *StHPL/EAS*, *StAOS2* and *StAOS3* in shoots throughout all time points (Figure 7A). The expression of *StAOS1*, *StHPL*, *StAOC* and *StJAZ/TIFY10a-like* was comparable to the control or slightly reduced. In roots, genes of the lipoxygenase cascade had similar expression patterns under treatment with NaCl (Figure 7B). The expression of *StAOS3* and *StHPL* noticeably increased, and the expression of *StAOS3* was maximal on the first day (100 times more than in the control sample), and displayed a declining trend for 14 days, while the expression pattern of *StHPL* was reversed—the maximal accumulation of transcripts was observed in 14 days. Other lipoxygenase cascade genes possessed smaller changes of expression dynamics in comparison with *StAOS3* and *StHPL*. The expression of *StHPL/EAS*, *StAOC* and *StLOX* was comparable to the control; the expression of *StDES*, *StAOS1*, *StAOS2* and *StJAZ/TIFY10a-like* tended to gradually decrease in 14 days.

An increase in *StAOS3* expression in roots rather than in shoots is in good agreement with the subcellular localization of this enzyme. On the basis of immunolocalization, it was shown that the specific signal was detected at amyloplasts and leucoplasts from the subterranean organs, but not at mesophyll chloroplasts [57], in contrast to other CYP74 enzymes (StAOS1, StAOS2 and StHPL) localized in chloroplasts [58]. In green parts, StAOS3 was found only in a subset of plastids, specifically amyloplasts and non-differentiated plastids in the stelar parenchyma [57]. Thus, in roots under salt stress, the biosynthesis of 9-AOS products by StAOS3 is activated. An increase in the expression of *StAOS3* in shoots during darkness (Figure 3) may indicate that the above-ground and underground parts become similar in a number of signs. The suppression of the expression of the negative regulator JAZ/TIFY of jasmonate signal transmission [45,46] is in good agreement with the data on the role of jasmonates in salt stress [59,60], since when the negative regulator is removed, the transcription of JA-induced genes is activated [61]. In addition, experimental data show that salt-tolerant varieties of tomatoes (close relatives of potatoes) have higher levels of jasmonates than salt-sensitive varieties [62].

**Figure 8:**
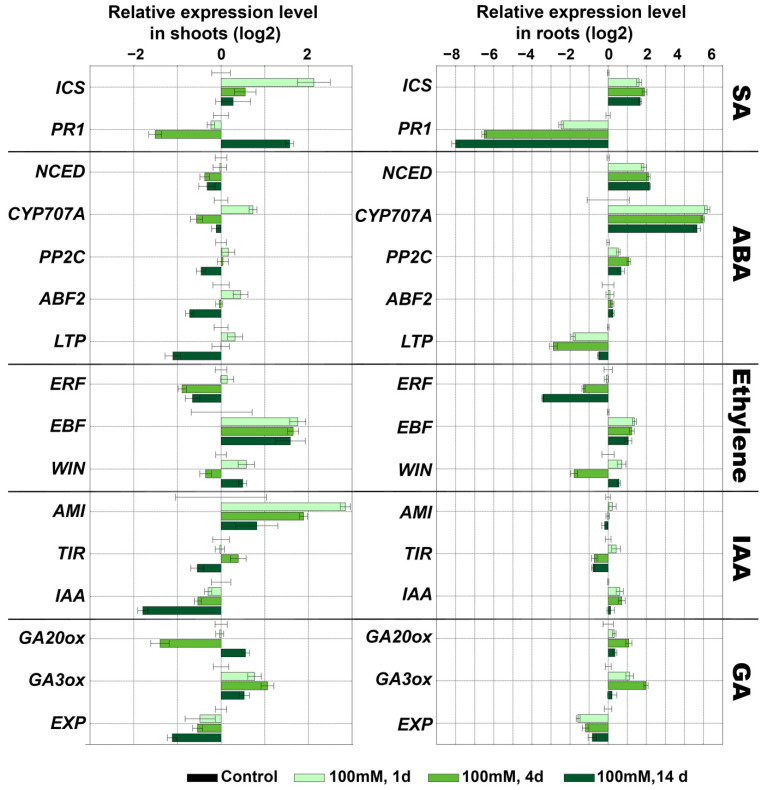
Changes in marker gene expression of other signaling pathways in response to treatment with NaCl (100 mM) in shoots and roots. Abbreviations: ICS: isochorismate synthase, PR1: pathogenesis-related protein 1, ERF: ethylene-inducible transcription factor, EBF: EIN3 (ETHYLENE-INSENSITIVE3) BINDING F-BOX1, WIN: wound-induced (WIN) protein, NCED: 9-*cis*-epoxycarotenoid dioxygenase, CYP707A: ABA-8′-hydroxylases, ABF2: ABRE-binding bZIP proteins, PP2C: serine/threonine phosphatase 2C, LTP: lipid transfer protein, GA20ox: gibberellin (GA) 20-oxidase, GA3ox: GA3 oxidase, EXP: expansins, AMI: indole-3-acetamide hydrolase, TIR: transport inhibitor response and IAA: indole-3-acetic acid inducible 28 (IAA28).

NaCl treatment caused a small accumulation of transcripts of the ICS gene (SA biosynthesis) in shoots (1.5–7 times) and roots (2–6 times), while significant changes in expression of marker genes of ABA signaling were observed only in roots (Figure 8). Gradually in 14 days after treatment with 25 and 50 mM NaCl and on the first day after treatment with 100 mM NaCl, the expression of NCED gene (ABA biosynthesis) was induced by up to 10 times more in comparison to the control. However, there was also a gradual accumulation of the CYP707A gene transcripts (ABA degradation) in 14 days after treatment with 25 and 50 mM (up to 80 and 40 times, respectively). Moreover, the expression of the CYP707A gene was maximal already on the first day after treatment with 100 mM NaCl. Additionally, the transcription of the PP2C gene (negative regulator of ABA signal transmission) and ABF2 transcription factor was slightly activated. Expression of the LTP gene (with an ABA-dependent expression pattern) decreased by an order of magnitude.

Salt treatment induced a gradual decrease in transcription of the ERF gene (ethylene-dependent gene) (from 1 to 0.5 in shoots and from 0.9 to 0.09 in roots) and an increase in transcription of the EBF gene (negative regulation of ethylene signaling) (up to 4 times in shoots; up to 3 times in shoots). Additionally, in roots, a decrease in the expression of the WIN gene (up to 2 times) was observed. In shoots, expression of this gene was comparable to the control. The data indicated a possible inhibition of ethylene signaling. In shoots, the expression of the AMI gene (auxin biosynthesis) increased by 2–14 times, while the expression of the IAA28 gene (repressor of IAA signaling) decreased by 2–8 times (Figure 8). Expression of the TIR1 gene slightly differed from the control values depending on the experimental point. In roots, the pattern of expression of these genes was opposite, but not significantly. The expression of genes of GA biosynthesis differed depending on the experimental point and had no clear picture. In the leaves at all three NaCl concentrations, at most points, the expression of the GA20ox gene decreased to 0.3, while the expression of the GA3ox gene increased to 2 (Figure 8 and Supplementary Figure S1). In the roots, on the contrary, at all three NaCl concentrations, at most points, an increase in the expression of the GA20ox gene to a value of 2 was observed. The expression of the GA3ox gene after treatment with 25 and 50 mM NaCl decreased to a value of 0.2 (Supplementary Figure S1), while after treatment with 100 mM NaCl it increased by 4 times.

Since the main organs of GA biosynthesis are considered to be young leaves and the expression of GA biosynthesis genes in the shoots was reduced in most samples (excluding GA3ox after treatment with 100 mM NaCl), this may indicate the inhibition of the GA pathway.

An increase in the expression of the ICS gene indicates its participation in adaptive regulation during salinity, and is consistent with previous studies [63,64]. As shown earlier, SA enhances the antioxidant system and the synthesis of osmolytes, and promotes plant photosynthesis under salt stress [65]. An increase in the expression of the NCED gene (ABA biosynthesis) is consistent with the literature data on the role of ABA as one of the key hormones in plant adaptation to salt stress [66,67]. Along with the activation of ABA biosynthesis, there is activation of auxin biosynthesis (in shoots), which plays an important role in the formation of lateral roots. High NaCl concentrations inhibit the development of lateral roots through ABA-coordinated polar auxin transport [68,69,70]. Apparently, the ABA-8’-hydroxylase gene is activated to regulate the level of ABA. This is necessary for functioning of the auxin system, as auxin transport is ABA-dose-dependent. The inhibition of the expression of genes of GA biosynthesis may be explained by switching to other signaling pathways. Additionally, regulating GA levels may be a mechanism of plant adaptation to growth inhibition due to salt stress. It corrects the growth rate in accordance with changing environmental conditions. Thus, genes associated with GA catabolism in Arabidopsis *(AtGA2ox7* [71] and rice *OsGA2ox5* [72]) increase plant resistance to salt by slowing growth. Along with the central players, ABA, *GA and* auxins participating in the regulation of adaptation and growth under salt stress, new players become involved, including enzymes of the lipoxygenase cascade (Figure 7). Thus, salt stress, being multifaceted and multicomponent, causes a significant response in signaling systems, provoking the solution of various tasks.

### 2.6. Activity of Lipoxygenase Cascade-Related Enzymes after Treatment with NaCl, Paraquat and Darkness

The activity of the target enzymes of the lipoxygenase cascade (LOX, CYP74s, etc.) in shoots and roots was analyzed as a result of accumulation of the corresponding oxylipins in plant extracts after incubation with linoleic and alpha-linolenic acids. Oxylipin composition is considered to reflect the physiological/functional state of a plant; therefore, the term ‘oxylipin signature’ has been put forward [73]. The internal standard was margaric acid (17:0), which is not converted during the lipoxygenase cascade. The following samples were analyzed by GC-MS: 1 day after treatment with 100 mM NaCl; 5 days after moving plants into the dark; and 4 h after treatment with 10^−6^ mM paraquat. These samples possessed the maximal expression of lipoxygenase cascade genes.

**Figure 9:**
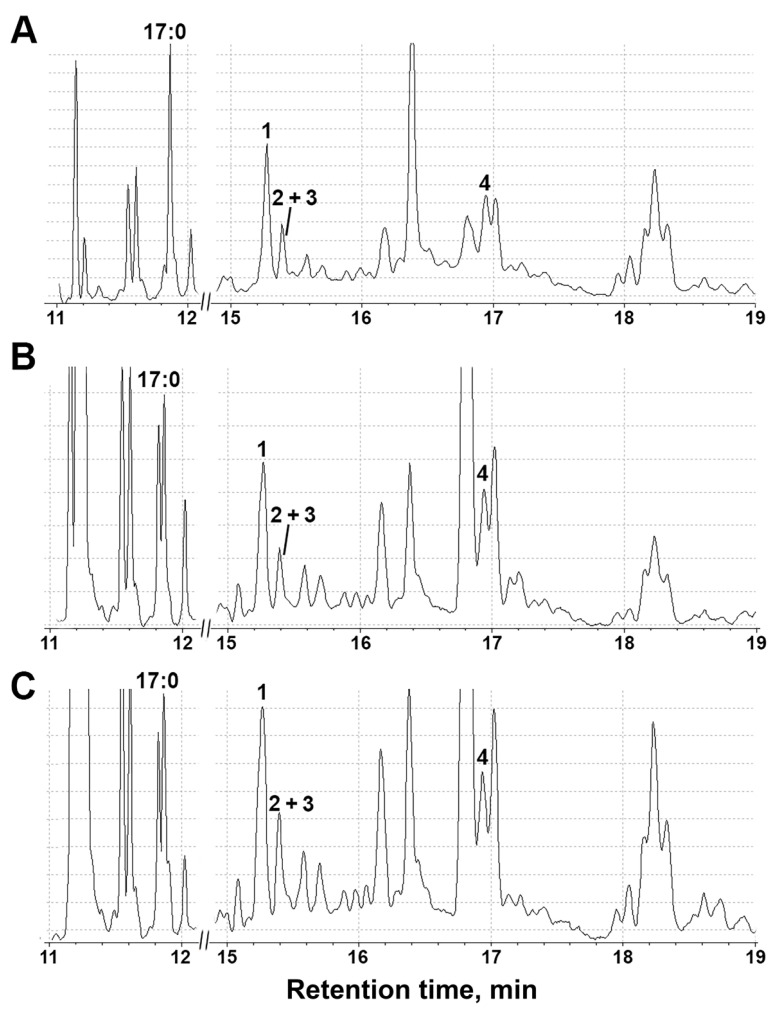
The total ion current (TIC) GC-MS chromatograms of lipoxygenase cascade products (Me/TMS) in *S. tuberosum* shoot extracts after incubation with linoleic and α-linolenic acids: (**A**) control plants; (**B**) plants during darkness; and (**C**) plants under oxidative stress. **1**: (9*S*,*10E*,12*Z*)-9-hydroxy-10,12-octadecadienoic acid (9-HOD, derivative of 9-LOX product); **2**: (9*S*,10*E*,12*Z*,15*Z*)-9-hydroxy-10,12,15-octadecatrienoic acid (9-HOT, derivative of 9-LOX product); **3**: 13-HOD + 13-HOT, **4**: 9,10-epoxy-11-hydroxy-12-octadecenoic acid (9-EAS product). 17:0: margaric acid (internal standard).

**Figure 10:**
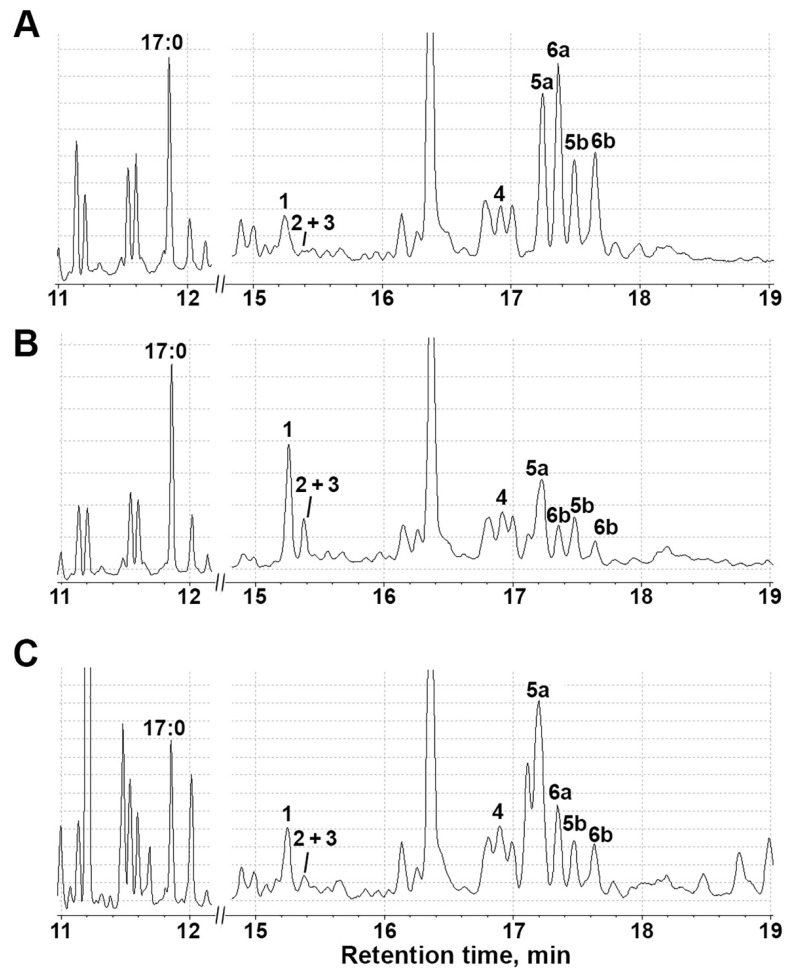
The TIC GC-MS chromatograms of lipoxygenase cascade products (Me/TMS) in *S. tuberosum* root extracts after incubation with linoleic and α-linolenic acids: (**A**) control plants; (**B**) plants under during darkness; and (**C**) plants after treatment with paraquat. **1**: 9-HOD (derivative of 9-LOX product); **2**: 9-HOT (derivative of 9-LOX product); **3**: 13-HOD + 13-HOT; **4**: 9,10-epoxy-11-hydroxy-12-octadecenoic acid (9-EAS product); **5a** and **5b:** *threo* and *erythro* isomers of 9,10-dihydroxy-12-octadecenoic acid (*vic*-diols formed the NaBH_4_ reduction in alpha-ketol, 9-AOS product of 9-HPOD conversion); **6a** and **6b:** *threo* and *erythro* isomers of 9,10-dihydroxy-12,15-octadecadienoic acid (*vic*-diols formed the NaBH4 reduction in alpha-ketol, 9-AOS product of 9-HPOT conversion).

In control shoots and roots, the transformation of linoleic and α-linolenic acids by lipoxygenases into the corresponding hydroperoxides, which further serve as substrates for the CYP74 enzymes, was observed. In shoots and roots, the main product of lipoxygenase conversion was linoleic acid 9-hydroperoxide (compound **1**). Moreover, its content in the shoots (Figure 9A) was twice as high as in the roots (Figure 10A). 9-Hydroperoxide of α-linolenic acid (compound **2**), as well as 13-hydroperoxides of linoleic and α-linolenic acid (compound **3**), were detected in a significantly smaller quantity. Their content in the leaves was three times higher than in the roots. Additionally, the roots contained a significant number of 9-AOS pathway products, as evidenced by the appearance of the corresponding peaks **5a/5b** and **6a/6b** in the chromatogram. Compounds **5a** and **5b** had the same mass spectral patterns, which are depicted in Supplementary Figure S2 and possessed M^+^ at *m*/*z* 472 (0.1%), [M—Me]^+^ at *m*/*z* 457 (0.5%), [M—MeO]^+^ at *m*/*z* 441 (2%), [M—C11/C18]^+^ at *m*/*z* 361 (11%), [M—C10/C18 + TMS]^+^ at *m*/*z* 332 (3%), [361—TMSOH]^+^ at *m*/*z* 271 (58%), [M—C10/C18]^+^ at *m*/*z* 259 (56%), [M—C1/C9]^+^ at *m*/*z* 213 (31%), *m*/*z* 155 (58%), *m*/*z* 129 (32%), *m*/*z* 109 (25%) [CH2 = O^+^—SiMe3] at *m*/*z* 103 (31%) and [SiMe3]^+^ *m*/*z* 73 (100%). The spectrum matched that of the product of NaBH_4_ reduction in the α-ketol, 9-hydroxy-10-oxo-12-octadecenoic acid (Me/TMS). Compounds **6a** and **6b** also possessed the identical mass spectra, which are depicted in Supplementary Figure S3. Mass spectra were similar to those of compounds **5a** and **5b** and corresponded to the product of NaBH_4_ reduction in the α-ketol, 9-hydroxy-10-oxo-12,15-octadecadienoic acid (Me/TMS). α-Ketols synthesized from 9-HPOD (**5a** and **5b**) and 9-HPOT (**6a** and **6b**) were present in equal quantities. Additionally, the control roots contained 9-oxononanoic acid (9-HPL product), while the control shoots did not. The products of the 9-EAS pathway, 9,10-epoxy-11-hydroxy-12-octadecenoic acid (compound **4**), were also found.

Analysis of GC-MS chromatograms showed that darkness led to the accumulation of all linoleate and linolenate hydroperoxides (9-HPOD, 9-HPOT, 13-HPOD and 13-HPOT) in roots (especially 9-HPOD). These data are consistent with the results on the expression of the StLOX gene, the number of transcripts of which increased in the roots by 16 times as compared to the control (Figure 3). The StLOX protein was localized in the underground parts (roots and tubers) of the potato, as evidenced by the additional proteomic analysis of lipoxygenases in individual organs (Supplementary Table S3). A decrease in α-ketols was observed (Figure 9B), especially derived from 9-HPOT (3 times compared to the control). In shoots, the number of hydroperoxides did not change due to darkness. Darkness led to the appearance of 9-oxononanoic acid, as well as the accumulation of 9,10-epoxy-11-hydroxy-12-octadecenoic acid (9-EAS product) in shoots. The accumulation of the 9-EAS product is consistent with the increased expression of the *StHPL/EAS* gene in shoots (Figure 3A). As we described earlier, the StHPL/EAS enzyme exhibits double hydroperoxide lyase/epoxyalcohol synthase activity [74].

Oxidative stress led to slight increase in the amount of 9-HPOD in shoots (Figure 9C). Moreover, there was an increase in 9-oxononanoic acid and 9,10-epoxy-11-hydroxy-12-octadecenoic acid (9-EAS product) synthesized from 9-HPOD, which is consistent with increased expression of the *StHPL/EAS* gene. In roots (Figure 10C), oxidative stress also led to a slight increase in the amount of 9-HPOD, as well as a change in the ratio of the number of alpha-ketols compared to the control. Alpha-ketol synthesized from 9-HPOD increased (**5a/5b**), while the peak of α-ketol synthesized from 9-HPOT became smaller (**6a/6b**) (Figure 10C).

Analysis of the oxylipin profile of shoot and root samples after NaCl treatment did not reveal a significant difference from the control (Supplementary Figure S4). Interestingly, the DES products were not detected in any sample, which was also the case for the 13-HPL products. The absence of these oxylipins among the analyzed products is due to the fact that they are involved in defense reactions during attacks of pathogens (biotic stress), such as infections caused by *Pectobacterium atrosepticum* [29].

## 3. Materials and Methods

### 3.1. Plant Growth Conditions and Stress

Potato (*Solanum tuberosum* L.) variety Zhukovskiy ranniy (Greenvale AP Ltd., Cambs, UK) was used in the present study. Potato plants were grown axenically in tubes in a growth chamber with a 16 h light/8 h dark cycle in Murashige and Skoog medium [75]. After 30 days of sowing, potato seedlings were subjected to several types of stress: darkness, salinity and herbicide toxicity.

Salinity was caused by NaCl treatments (25, 50 and 100 mM). After 1, 4 and 14 days, the plants were harvested for further analyses. Herbicide toxicity was investigated using paraquat (1,1-dimethyl-4,4-bipyridinium dichloride) (10^−5^ and 10^−^^6^ M). After 1, 2, 4 and 6 h, the plants were harvested for further analysis. Darkness was ensured by transferring 20-day-old potato seedlings into the dark and maturing them there. Samples for analysis were taken on days 1, 2, 3, 5 and 7. A minimum of three biological replicates were used for each time point, and each biological replicate comprised material pooled from several plants.

### 3.2. RNA Extraction and cDNA Synthesis

Potato shoots (stems including their appendages, leaves and lateral buds) and roots were ground to a fine powder in liquid nitrogen, and total RNA samples were then extracted using the RNeasy Plant Mini kit (Qiagen, Hilden, Germany) with the processing step of DNase according to the manufacturer’s instructions. RNA quantity and quality were confirmed with a NanoDrop ND-1000 spectrophotometer (NanoDrop, Hampton, NH, USA) and 1% agarose gel electrophoresis. Total RNA (1 μg) was converted to cDNA with M-MuLV Reverse Transcriptase (Fermentas, Vilnius, Lithuania), according to the standard protocol from the manufacturer.

### 3.3. Quantitative Real-Time PCR (qRT-PCR)

Quantitative real-time PCR was carried out on a CFX96 Touch Real-Time PCR Detection System (Bio-Rad, Hercules, CA, USA) using 2.5× EVA Green master mix (Syntol, Moscow, Russia). Primers were designed using the Vector NTI Advance 11.5 program (Invitrogen, Waltham, MA, USA), according to the gene sequences in the GenBank and Phytozome Database. The specific primer pairs are shown in Supplementary Table S1. The PCR mixture contained 1× EVA Green master mix, 0.4 µM of each forward and reverse primer, cDNA and DNase free water for a total volume of 10 μL. The thermal profile of the real-time system was one step at 95 °C for 2 min, followed by 40 cycles at 94 °C for 10 s (denaturation) and at 60 °C for 15 s (annealing), 72 °C for 30 s (extension), followed by adding a 60–95 °C melting curve to confirm specificity of the products. From each of three biologically independent cDNA samples, two independent technical replications were performed, and averaged for further calculations. Relative transcript abundance calculations were performed using the CFX Maestro software (Bio-Rad, Hercules, CA, USA). In the work, the normalized gene expression (ΔΔCq) was calculated. The significance of the differences in results was determined using Student’s *t*-test. *p* < 0.05 was considered statistically significant. The data are presented as means ± SD.

### 3.4. Bioinformatics Methods

Amino acid and nucleotide sequences of CYP74 enzymes of *S. tuberosum* were taken from NCBI and Phytozome databases for phylogenetic analysis and illustrations of exon–intron organization genes. The multiple alignments of CYP74 amino acid sequences were carried out with MEGA7 software [76]. The phylogenetic tree of CYP74s was inferred using the maximum likelihood method based on the Poisson correction model [77]. The resulting phylogenetic data and gene characteristics were visualized using the Gene Structure Display Server (GSDS) [78].

The promoter sequences (1 kb upstream regions to the start codon) of target genes were retrieved from the potato genome sequence v4.03 from Phytozome database (http://www.phytozome.net/ accessed on 10 January 2022). The tools PlantCare (http://bioinformatics.psb.ugent.be/webtools/plantcare/html/ accessed on 10 January 2022 [79] and PLACE (https://www.dna.affrc.go.jp/PLACE/?action=newplace accessed on 10 January 2022) were used for the identification of *cis*-regulatory elements in promoters of *S. tuberosum* CYP74 genes.

### 3.5. Profiling of S. tuberosum Oxylipins under Abiotic Stresses

The *S. tuberosum* samples of tissues (shoots and roots, 2 g fresh weight) were homogenized in liquid nitrogen. After adding 4 mL of ice-cold 50 mM Tris-HCl buffer, pH 7.0, the samples were filtered through cheesecloth and incubated with 150 µg linoleic, 150 µg α-linolenic and 75 µg margaric (internal standard) acids at 23 °C for 30 min under continuous oxygen bubbling. The reaction mixture was acidified to pH 6.0 and the reaction products were extracted with a 10 mL mixture of ethyl acetate/hexane (1:1, by volume) as described previously [47]. Then, the resulting products were methylated with ethereal diazomethane and trimethylsilylated with pyridine/hexamethyldisilazane/trimethylchlorosilane (1:1:1, by volume) mixture at 23 °C for 30 min. Additionally, the products were reduced with NaBH_4_, and then methylated and trimethylsilylated. The silylation reagents were evaporated in vacuo, and dissolved in 100 µL of hexane. The obtained product derivatives (methyl esters/trimethylsilyl derivatives (Me/TMS)) were analyzed by GC–MS.

The GC–MS analyses were performed using a Shimadzu QP2020A mass spectrometer connected to Shimadzu GC-2010 Plus gas chromatograph equipped with a Macherey-Nagel Optima-5-MS (5% phenyl 95% methylpolysiloxane) fused capillary column (length, 30 m; ID 0.25 mm; film thickness, 0.25 μm) (Shimadzu, Kyoto, Japan). Helium at a flow rate of 30 cm/s was used as the carrier gas. Injections were performed in the split mode using an initial column temperature of 120 °C, with injector temperature of 230 °C. Then, the column temperature was raised at 10 °C/min until 240 °C. Electron impact ionization (70 eV) was used.

## 4. Conclusions

In higher plants, the most common C18 polyene fatty acids are 18:1 (oleate), 18:2 (linoleate) and 18:3 (α-linolenate) [80]. The suppression of the expression of a number of genes of the lipoxygenase cascade, in addition to the redistribution of signaling functions, may be due to the transfer of the main substrates (C18 fatty acids) from the biosynthesis of oxylipins to the formation of protective biopolymers (cutin and suberin), which are also derivatives of fatty acids [81]. This is confirmed by the literature data showing that the monomeric composition of cutin or suberin can vary greatly depending on the type of plant, organ and stage of development. In Arabidopsis, for example, the cutin of leaves and stems has an unusually high content of dicarboxylic acids derived from 18:2 [82], while the cutin of flowers is mainly composed of dihydroxy acids derived from 16:0 [83]. In addition, there are other protective biopolymers consisting of aliphatic and aromatic hydrocarbons such as cutane. It is present in some plants as an additional cuticle fraction [84], with a ratio of 18:2 apparently preferred as a building block over 16:0 [85].

Interestingly, both under abiotic and biotic stresses, activation of the 9-LOX pathway was detected. However, in the case of biotic stress in cultured potato cells treated with elicitor from *Phytophthora infestans*, the accumulation of colneleic acid (a product of the 9-DES branch of the LOX pathway) was detected [86]. A similar result was observed in tobacco (*Nicotiana tabacum*) plants, where the impact of the elicitor or pathogen led to the accumulation of colneleic and colnelenic acids [87]. Thus, different types of stress factors can lead to the same intermediate products (in this case, 9-HPOD), which are then utilized by different CYP74 enzymes. Significant changes in the profiles of oxylipins were related to the transformation of 9-HPOD in 9-AOS and 9-EAS pathways (Figure 9 and Figure 10). Since all CYP74 enzymes of potato have been characterized, 9-EAS products can be formed only by StHPL/EAS, which also exhibits HPL activity, and is able to convert not only 9- but also 13-hydroperoxides [74].

The obtained data on the lipoxygenase system complement the multiplicity of interactions between signaling systems that directly or indirectly link biosynthesis, perception and signaling of various phytohormones. The final formation of oxylipins will depend not only on the activation of certain genes, but more importantly the presence of specific CYP74 enzymes (AOS, DES, HPL, EAS, etc.) and the availability of substrates, since some CYP74 enzymes have cytosolic localization and some are membrane-associated. Therefore, in violation of the integrity of tissues, de novo formation of oxylipins can occur without the activation of certain genes, but only due to the availability of the substrate. Along with this, it is obvious that the signaling pathways do not function separately, but are cross-regulated, leading to plant adaptation to current conditions. Preactivation of one resistance mechanism can increase plant resistance to a number of factors. The results obtained during this study expand knowledge of a vast and diverse group of plant metabolites, oxylipins, which not only participate in the defense mechanisms of plants, but also determine the number of valuable organoleptic properties of plants. For example, the volatile C6 and C9 compounds (green leaf volatiles) of the hydroperoxide lyase branch impart aromas and flavors to many vegetables and fruits. Further study of already known key players and the identification of new components in phytohormone interaction networks will expand understanding of the mechanisms underlying the delicate balance of plant growth and development and their defense responses.

## Figures and Tables

**Table 1:** *Cis*-elements detected in 1 kbp upstream region of CYP74 genes of *S. tuberosum*: MRE: MYB-recognition element; SORLIP1AT: sequences over-represented in light-induced promoters; ABRE: abscisic acid responsiveness; ACGTATERD1: ACGT sequence required for etiolation-induced expression of erd1 (early responsive to dehydration); ABRELATERD1: ABRE-like sequence required for etiolation-induced expression of erd1; WBOXATNPR1: W-box found in promoter of Arabidopsis NPR1 gene; GT1GMSCAM4: GT-1 motif found in the promoter of soybean (*Glycine max*) CaM isoform, SCaM-4; GARE1OSREP1: gibberellin-responsive element (GARE) found in the promoter region of a cysteine proteinase (REP-1) gene in rice.

Functions of *cis*-Elements	*Cis*-Element	Sequence	Tools Used
Light responsiveness	Box 4	ATTAAT	PlantCARE
	G-Box	CACGTC, CACGTT, TACGTG	PlantCARE
	GT1-motif	GGTTAA	PlantCARE
	TCT-motif	TCTTAC	PlantCARE
	MRE	AACCTAA	PlantCARE
	I box, SORLIP1AT	GCCAC	PLACE
Abscisic acid	ABRE	ACGTG, CACGTG, CACGTA	PlantCARE
Etiolation-induced	ACGTATERD1	ACGT	PLACE
	ABRELATERD1	ACGTG	PLACE
Pathogen and salt-induced	GT1GMSCAM4	GAAAAA	PLACE
Salicylic acid	TCA-element	CCATCTTTTT	PlantCARE
	WBOXATNPR1	TTGAC	PLACE
Auxin-responsive element	TGA-element	AACGAC	PlantCARE
	NTBBF1ARROLB	ACTTTA	PLACE
Gibberellin- responsive	MYB	CAACCA, TAACCA	PlantCARE
	P-box	CCTTTTG	PlantCARE
	GARE1OSREP1	TAACAGA	PLACE
Repressor of the gibberellin pathway	WRKY71OS	TGAC	PLACE

## Data Availability

All the data are available in the manuscript and Supplementary Materials.

## Supplementary Materials

The following supporting information can be downloaded at: https://www.mdpi.com/article/10.3390/plants11050683/s1, Figure S1: Changes in marker gene expression of other signaling pathways in response to treatment with NaCl (25 and 50 mM) in shoots and roots; Figure S2: The electron impact mass spectrum of compounds 5a (vic-diol) and the corresponding mass fragmentation scheme; Figure S3: The electron impact mass spectrum of compounds 6a (vic-diol) and the corresponding mass fragmentation scheme; Figure S4: The total ion current (TIC) GC-MS chromatograms of lipoxygenase cascade products (Me/TMS) in *S. tuberosum* shoot extracts after incubation with linoleic and α-linolenic acids: (A) shoots after NaCl treatment; (B) roots after NaCl treatment; Table S1: Primers for qRT-PCR amplification.; Table S2: List of marker genes for different hormonal systems; and Table S3: Identified lipoxygenase (StLOX) in underground potato organs.
