# Supplementary material for: Gene Expression Analysis of Potato (Solanum tuberosum L.) Lipoxygenase Cascade and Oxylipin Signature under Abiotic Stress
Source: Plants (Basel). 2022 Mar 2;11(5):683. doi: 10.3390/plants11050683 (PMC8912661; doi:10.3390/plants11050683)
Supplement: Supplementary file 1 [file plants-11-00683-s001.zip › plants-1572832-supplementary.pdf]

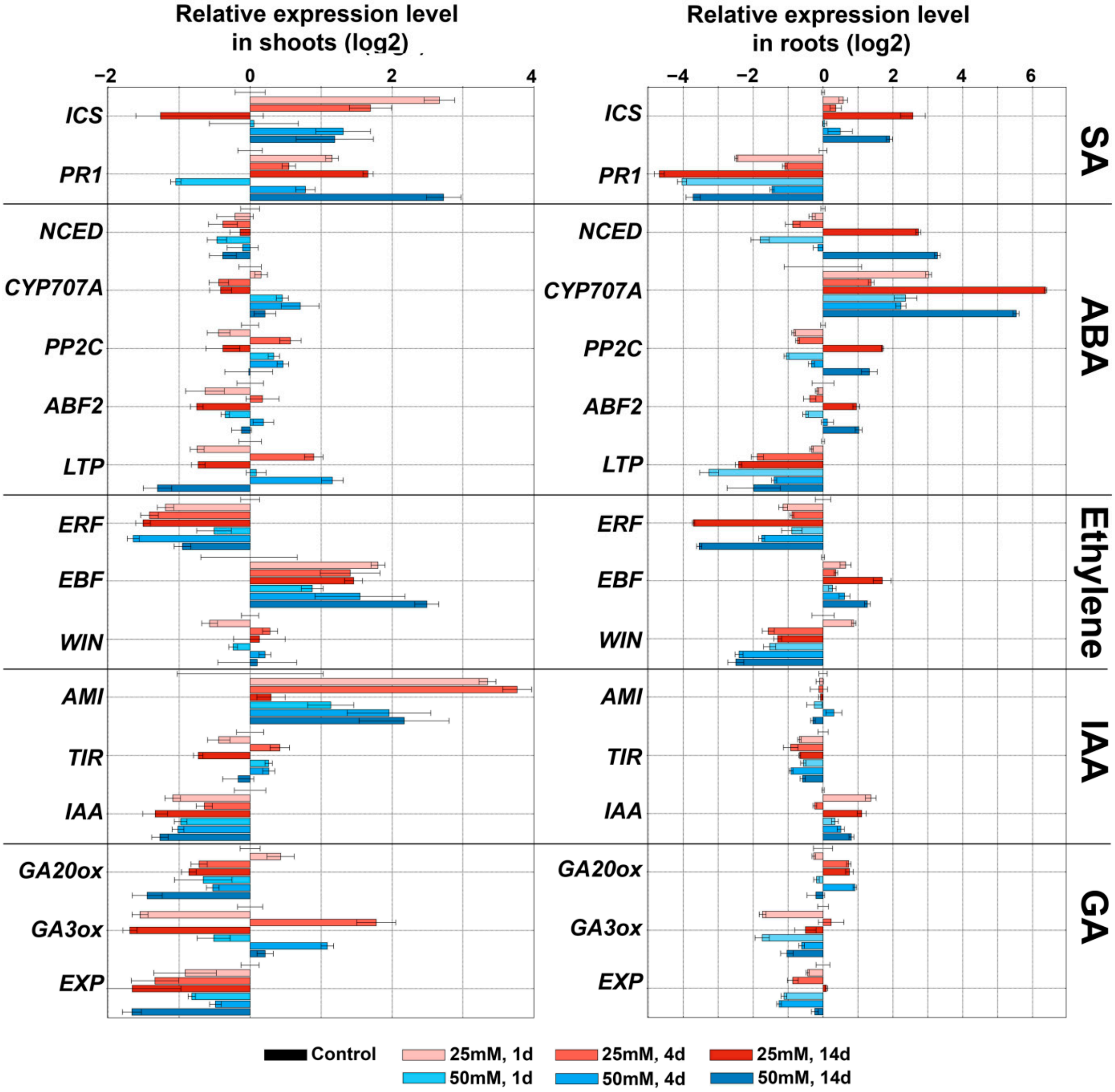

Supplementary Figure S1. Changes in marker genes expression of other signaling pathways in response to treatment with NaCl 25 mM (red) and 50 mM (blue) in shoots and roots. Abbreviations: ICS (isochorismate synthase), PR1 (pathogenesis-related protein 1), ERF (ethylene-inducible transcription factor), EBF (EIN3 (ETHYLENE-INSENSITIVE3) BINDING F-BOX1), WIN (wound-induced (WIN) protein), NCED (9-cis-epoxycarotenoid dioxygenase), CYP707A (ABA-8′-hydroxylases), ABF2 (ABRE-binding bZIP proteins), PP2C (serine/threonine phosphatase 2C), LTP (lipid transfer protein), GA20ox (gibberellin (GA) 20-oxidase), GA3ox (GA3 oxidase), EXP (expansins), AMI (indole-3-acetamide hydrolase), TIR (transport inhibitor response), IAA (indole-3-acetic acid inducible 28 (IAA28)).

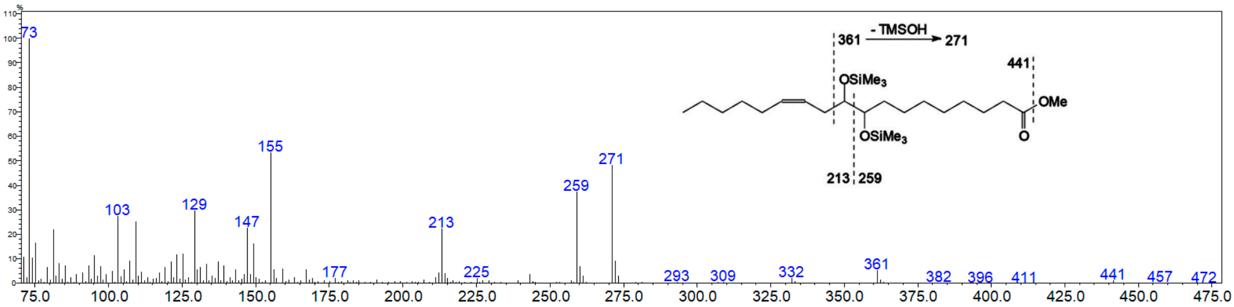

Supplementary Figure S2. The electron impact mass spectrum of compounds 5a (vic-diol) and the corresponding mass fragmentation scheme. Compound 5b has the identical spectrum.

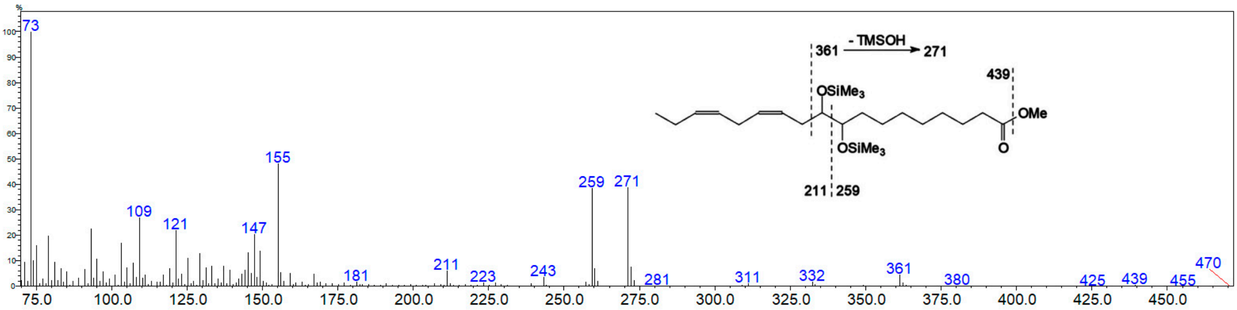

Supplementary Figure S3. The electron impact mass spectrum of compounds 6a (vic-diol) and the corresponding mass fragmentation scheme. Compound 6b has the identical spectrum.

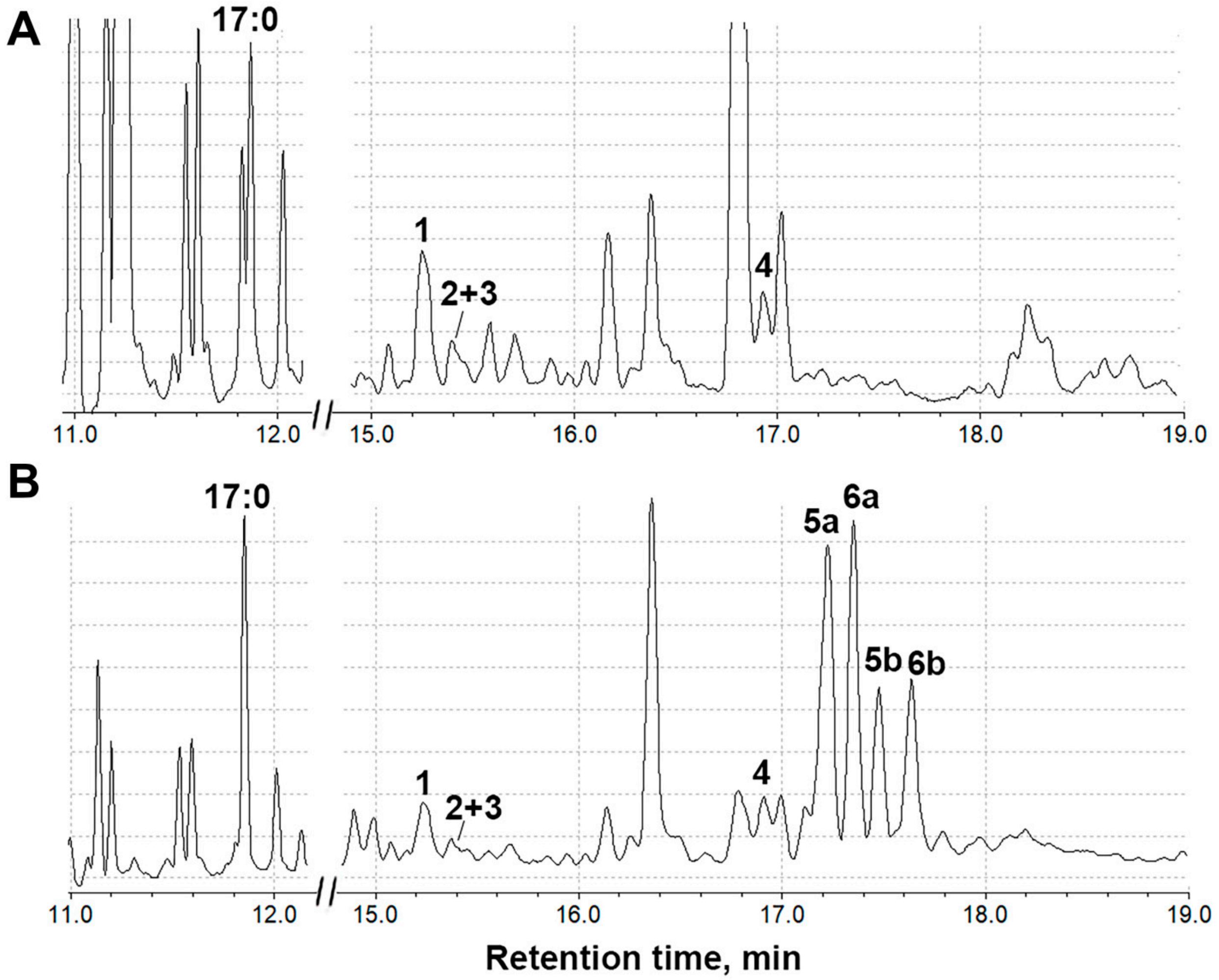

Supplementary Figure S4. The total ion current (TIC) GC-MS chromatograms of lipoxygenase cascade products (Me/TMS) in *S. tuberosum* shoots extracts after incubation with linoleic and α-linolenic acids: (A) shoots after NaCl treatment; (B) roots after NaCl treatment. **1** – 9-HOD (derivative of 9-LOX product); **2** – 9-HOT (derivative of 9-LOX product); **3** – 13-HOD + 13-HOT, **4** – 9,10-epoxy-11-hydroxy-12-octadecenoic acid (9-EAS product); **5a** and **5b** – *threo* and *erythro* isomers of 9,10-dihydroxy-12-octadecenoic acid (*vic*-diols formed the NaBH4 reduction of alpha-ketol, 9-AOS product of 9-HPOD conversion), **6a** and **6b** – *threo* and *erythro* isomers of 9,10-dihydroxy-12,15-octadecadienoic acid (*vic*-diols formed the NaBH4 reduction of alpha-ketol, 9-AOS product of 9-HPOT conversion).

Supplementary Table S1. Primers for qRT-PCR amplification.

	Gene name	Forward primer	Reverse primer
**LOX pathway**	*StLOX*	GTAGACCACCAACACGAACAGATCC	CAGGGCATGTAATTCAGGGAGG
	StAOS1	CCATCGCCACCTGTTAAACAAG	CCGCCTGTGAGATCAGTGGAG
	StAOS2	CCCAAATTCCATGAAACTTATACAGAG	GACCGAGAGTGAGTACAGGATGAAG C
	StAOS3	CCTATTTCGATTGTTTTGTGAGGGG	CTCCAAGTTTCTCCGCTTCATC
	StHPL	CTGAACCTAAACATGCCCAGATTAAG	GCCGAGGAGAGTGAGGGAGAAG
	StDES	CCCTCATCTCTTCAGGTTTGTGG	GGTGGGCGAAGTCTCAATGTCTC
	StHPL/EAS	CTTGCAGGATTCAACGCTTATGG	CACCTTCCTCCTTGATGATCGTC
	StJaz	CAAGTGATTCATCGTCGTCATCGTC	GAAAATCATCAAAAACAATGACTTGT CC
	StAOC	GCTCAATGAACGTGACCGTGGAAG	CGATCTCCTTTCTTCTCTTCTTCGTG
**SA**	*StICS*	GGTACTTGCTCGTAGCACCAGAGTTG	GCTGAGGCGGTCCCGATGAAATAG
	StPR1	CATCTCATTGTTACTCACTTGTCTCATGG	GAATTGGCATAATTTTGTGCTCGGGAT G
**Ethylene**	*StERF*	GGATGGTCGCCGTTTAGTTTCAC	CCGCTGTCTAACGCCTCTATAATGTC
	StEBF	GCCGTGACTGACATAGTCCTTATTGG	CAGAACAGCTTCAAGTTTGGACAACC
	StWIN	GCCGTGACTGACATAGTCCTTATTGG	CAGAACAGCTTCAAGTTTGGACAACC
**ABA**	*StABF2*	GACCACAGCAACTGCAACAGAAC	CTGCCCAACTGCCCACTATTTG
	StNCED2	GCGAACTACAAAACCCTCTTCC	CCCCGTTAATGCAATTAGGG
	StPP2C	CGAGGTGGAGAAGGAAGAAACTC	CACTGTGGAGTCTGAAGCTCGC
	StC707A	GTGTTTAAGCTCTTTGATTGTGGTG	CTGGGGTTTTGTGAGTAAAGCTG
	StLtp1	GTCCTCTCGGAGGCTGTTGTG	GGGAGACTAGCAGCTTTGCCC
**GA**			
	StGA3ox	GCCCTACAACTAAATTCTTACCCG	GGTACAACCCGTTTGACAATATGTG
	StGA20ox	CAAAGCTACCTTGGAAAGAGACACTC	GAAGTGGCTCTTCTCTACGCCTAG
	StEXP7	GCCTAATGATAATGGTGGTTGGTG	CATAACCTTCGTAATGTCACCTGC
***Auxin*, IAA**	*StAMI*	GAGTACCTGGAGGATCTTCAAGTGG	CATCCAACTGTATCAAAACTTTGTGC
	StTIR1	GAAGTGGAGGACCTTAGTGGGC	GCACGATTTATCCGAAGAGTTCTC
	StIAA28	GGGTCTTTTCAGAAACTGTTGATTTG	CTTCCTGAATGATCTCACTGGTGG
**Housekeeping genes**	*STTub*	GTTACTTGCTGTTTGAGATTCCCTG	CTGTCAGGTAACGTCCATGACG
	STEF	CTTCCTACCTCAAGAAGGTTGGTTAC	CTTGTCTGTTGGTCTCTTGGGC
	STGAP	GGTTAAGGATGAGAAGACCCTTC	GCGGAGATCACAACCTTCTTG
	STUbi	GACAATGTTAAGGCTAAGATTCAAGAC	GTGGACTCCTTCTGGATGTTGTAG
	STAct	GTGAGTCACACTGTCCCTATCTATG	CAGTGGTGGTGAACATATAACCTCT
	STATP	GGTAAAGGAGGAGTTGGGAAGAC	CAAGGGCAAATAATGGAGAATAC

Supplementary Table S2. List of marker genes for different hormonal systems, the expression of which was analyzed in the present study.

	Gene designation in the article (in the figures)	Gene name	Function/Role
**SA**	ICS	Isochorismate synthase	SA biosynthesis
	PR1	Pathogenesis-related protein 1	Defense reaction
**Ethylene**	ERF	Ethylene-inducible transcription factor	Activation of the expression of ethylene-induced genes
	EBF	**E**IN3 (ETHYLENE-INSENSITIVE3) **B**INDING **F**-BOX1	Negative regulator of ethylene-dependent signaling
	Win	Wound-induced (Win) protein	Defense reaction
**ABA**	NCED	9-cis-epoxycarotenoid dioxygenase	ABA biosynthesis
	C707A3	ABA-8′-hydroxylases	ABA degradation
	ABF2	ABRE-binding bZIP proteins (transcription factor)	ABA signal transmission
	PP2C	serine/threonine phosphatase 2C	Negative regulator of ABA signal transmission
	LTP	Lipid Transfer Protein	Lipid Transfer, membrane stabilization
**GA**	GA20ox	GA20-oxidases	Gibberelin biosynthesis
	GA3ox	GA3 oxidase	Gibberelin biosynthesis
	EXP	Expansins	Loosening of the cell wall
**Auxin (IAA)**	AMI	Indole-3-acetamide hydrolase	Auxin biosynthesis
	TIR	Transport inhibitor response	Auxin signal transmission
	IAA	Indole-3-acetic acid inducible 28 (IAA28)	Repressor of IAA signaling

Supplementary Table S3. Identified lipoxygenase (StLOX) in underground potato organs. The matched peptides in the protein sequence are highlighted in red.

Organ	Protein	Score	Protein sequence
roots and tuber	Q43190 (LOX14_SOLTU)<br>Lipoxygenase<br>Mr 96909<br>pI 5.50	597	**1** MLGQIVGGLI GGHHDSKKVK GTVVMMKK**NA LDFTDLAGSL TDK**IFEALGQ<br>**51** KVSFQLISSV QSDPANGLQG KHSNPAYLEN FLFTLTPLAA GETAFGVTFD<br>**101** WNEEFGVPGA FIIKNTHINE FFLKSLTLED VPNHGKVHFV CNSWVYPSFR<br>**151** YKSDR**IFFAN QPYLPSETPE LLR**KYRENEL LTLRGDGTGK REAWDRIYDY<br>**201** DVYNDLGNPD QGKENVRTTL GGSADYPYPR RGRTGRPPTR TDPKSESR**IP**<br>**251 LILSLDIYVP RDER**FGHLKM SDFLTYALK**S IVQFILPELH ALFDGTPNEF**<br>**301 DSFEDVLR**LY EGGIR**LPQGP LFK**ALTDAIP LEMIRELLRT DGEGILRFPT<br>**351** PLVIKDSKTA WRTDEEFAR**E MLAGVNPIII SR**LQEFPPKS K**LDPEAYGNQ**<br>**401 NSTITAEHIE DKLDGLTVDE AMNNNK**LFIL NHHDVLIPYL RRINTTTTKT<br>**451** YASR**TLLFLQ DNGSLKPLAI ELSLPHPDGD QFGVTSKVYT PSDQGVESSI**<br>**501 WQLAK**AYVAV NDSGVHQLIS HWLNTHAVIE PFVIATNRQL SVLHPIHKLL<br>**551** YPHFRDTMNI NAMARQILIN AGGVLESTVF PSK**FAMEMSA VVYKDWVFPD**<br>**601 QALPADLVK**R GVAVEDSSSP HGVR**LLIEDY PYAVDGLEIW SAIK**SWVTDY<br>**651** CSFYYGSDEE ILKDNELQAW WKELREVGHG DKKNEPWWPE METPQELIDS<br>**701** CTTIIWIASA LHAAVNFGQY PYAGYLPNRP TVSRRFMPEP GTPEYEELKK<br>**751** NPDKAFLK**TI TAQLQTLLGV SLIEILSR**HT TDEIYLGQRE SPEWTKDKEP<br>**801** LAAFDKFGKK LTDIEKQIIQ RNGDNILINR **SGPVNAPYTL LFPTSEGGLT**<br>**851 GK**GIPNSVSI
tuber	Q43190_SOLTU<br>Lipoxygenase<br>Mr 96909<br>pI 5.50	303	**1** MLGQIVGGLI GGHHDSKKVK GTVVMMKK**NA LDFTDLAGSL TDK**IFEALGQ<br>**51** KVSFQLISSV QSDPANGLQG KHSNPAYLEN FLFTLTPLAA GETAFGVTFD<br>**101** WNEEFGVPGA FIIKNTHINE FFLKSLTLED VPNHGKVHFV CNSWVYPSFR<br>**151** YKSDR**IFFAN QPYLPSETPE LLR**KYRENEL LTLRGDGTGK REAWDRIYDY<br>**201** DVYNDLGNPD QGKENVR**TTL GGSADYPYPR** RGRTGRPPTR TDPKSESR**IP**<br>**251 LILSLDIYVP R**DERFGHLK**M SDFLTYALK**S IVQFILPELH ALFDGTPNEF<br>**301** DSFEDVLRLY EGGIRLPQGP LFK**ALTDAIP LEMIR**ELLRT DGEGILRFPT<br>**351** PLVIKDSKTA WRTDEEFAR**E MLAGVNPIII SR**LQEFPPKS KLDPEAYGNQ<br>**401** NSTITAEHIE DKLDGLTVDE AMNNNK**LFIL NHHDVLIPYL R**RINTTTTKT<br>**451** YASRTLLFLQ DNGSLKPLAI ELSLPHPDGD QFGVTSK**VYT PSDQGVESSI**<br>**501 WQLAK**AYVAV NDSGVHQLIS HWLNTHAVIE PFVIATNRQL SVLHPIHKLL<br>**551** YPHFRDTMNI NAMARQILIN AGGVLESTVF PSKFAMEMSA VVYK**DWVFPD**<br>**601 QALPADLVK**R GVAVEDSSSP HGVRLLIEDY PYAVDGLEIW SAIKSWVTDY<br>**651** CSFYYGSDEE ILKDNELQAW WKELREVGHG DKKNEPWWPE METPQELIDS<br>**701** CTTIIWIASA LHAAVNFGQY PYAGYLPNRP TVSRRFMPEP GTPEYEELKK<br>**751** NPDKAFLKTI TAQLQTLLGV SLIEILSRHT TDEIYLGQRE SPEWTKDKEP<br>**801** LAAFDKFGKK LTDIEKQIIQ RNGDNILINR **SGPVNAPYTL LFPTSEGGLT**<br>**851 GK**GIPNSVSI
